# Emerging New Pathways in Malignant Neoplasms and Neurodegenerative Disorders: Perspectives for Therapeutics

**DOI:** 10.3390/cells15131177

**Published:** 2026-06-29

**Authors:** Wolf Wrasidlo, Eliezer Masliah

**Affiliations:** 1 Radiation Medicine and Applied Sciences Department, University of California, San Diego, CA 92093, USA; 2 Department of Neurosciences, University of California, San Diego, CA 92093, USA

**Keywords:** neurodegeneration, oncogenesis, reciprocal genes, cell senescence, lysosomal pathways, Alzheimer’s disease, lung carcinoma

## Abstract

**Highlights:**

**What are the main findings?**

- The inverse cancer–neurodegeneration relationship is not uniform; it varies by cancer type and neurodegenerative diagnosis, with stronger inverse patterns for AD and PD/Lewy body spectrum disorders, reduced overall cancer prevalence reported in FTLD, and melanoma representing an important exception in PD.
- Cancer and neurodegeneration may reflect divergent aging-related pathway trajectories, involving PI3K–AKT–mTOR, p53/DNA damage responses, IGF/MAPK, BCL-2, proteostasis/autophagy, senescence/SASP signaling, and transcriptomic/genetic programs that can shift toward malignant proliferation or neurodegenerative vulnerability.

**What are the implications of the main findings?**

- Pathology-resolved and cancer subtype-specific analyses are needed to distinguish true biological inverse relationships from survival bias, diagnostic ascertainment, treatment effects, and mixed dementia pathology.
- The cancer–neurodegeneration interface may reveal therapeutic opportunities, including oncology-derived kinase inhibitors, lysosomal modulators, epigenetic modulators, senolytics, and transcriptomic drug-repurposing strategies, but interventions must account for the trade-off between enhancing neuronal survival and increasing oncogenic risk.

**Abstract:**

Neurodegenerative disorders such as Alzheimer’s disease (AD) and malignant neoplasms are among the most prevalent age-associated diseases worldwide. Although cancer is characterized by uncontrolled proliferation, resistance to apoptosis, and metabolic reprogramming, AD and other neurodegenerative disorders such as Lewy body disease (LBD) including Parkinson’s Disease (PD) and fronto-temporal lobar degeneration (FTLD) are defined by synaptic dysfunction, neuronal loss, neuroinflammation, and impaired proteostasis with misfolded protein aggregates. Despite these contrasting phenotypes, converging epidemiological and molecular data support an inverse relationship between cancer and neurodegenerative disorders, whereby a history of cancer is associated with reduced AD risk, whereas AD is linked to a lower incidence of multiple malignancies. These observations suggest that oncogenesis and neurodegeneration may represent divergent outcomes of shared biological processes dysregulated during aging. This conundrum likely reflects differential regulation of core cellular pathways governing cell survival, stress responses, metabolism, and genomic integrity but could also reflect the differential influence of aging pathways and secreted growth factors. Pro-survival and proliferative signaling pathways commonly activated in cancer, including PI3K–AKT–mTOR signaling, altered p53 function, enhanced DNA damage tolerance, and anabolic metabolism, are often impaired in AD, LBD and FTLD, where neurons exhibit heightened vulnerability to stress, mitochondrial dysfunction, defective autophagy, and activation of pro-apoptotic cascades. Conversely, tumor-suppressive mechanisms that restrain proliferation may protect against malignancy but increase susceptibility to degeneration in post-mitotic neurons. Aging-related processes such as cellular senescence, immune dysregulation, and loss of proteostasis may further exert divergent effects in oncogenesis and neurodegeneration. This review aims to clarify associations between specific cancer types and neurodegenerative disorders, examine shared and opposing selected molecular mechanisms linking specific cancers and neurodegeneration, and contextualize these relationships within broader aging pathways (e.g., cell senescence, proteostasis). By integrating epidemiological, mechanistic, and therapeutic perspectives, we highlight unifying biological principles and translational opportunities at the intersection of cancer, neurodegeneration, and aging.

## 1. Introduction

**Figure 1:**
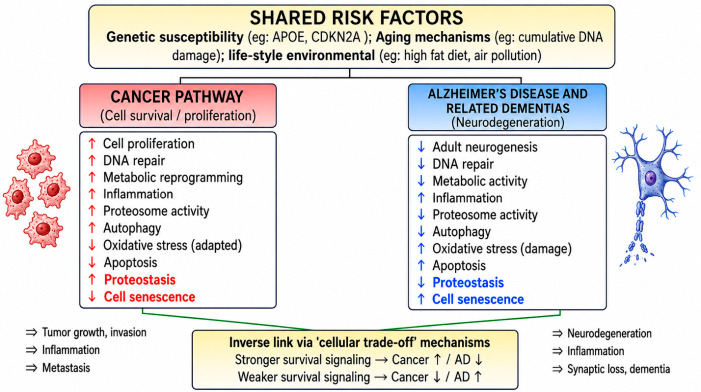
**Shared risk factors in cancer and Alzheimer’s disease and related dementias lead to divergent cellular outcomes**. Shared determinants, including genetic susceptibility, aging-related mechanisms, lifestyle and environmental exposures, and tissue microenvironmental conditions, may exert opposing biological effects in cancer and neurodegenerative disorders. In cancer, these influences are associated with increased cell proliferation, enhanced DNA repair, metabolic reprogramming, preserved proteostasis, reduced apoptosis, and reduced cellular senescence, thereby supporting tumor growth and invasion. In Alzheimer’s disease and related dementias, similar upstream risk factors are associated with reduced neurogenesis, impaired DNA repair and proteostasis, altered metabolism, increased oxidative stress, inflammation, apoptosis, and cellular senescence. This model illustrates an inverse link based on cellular trade-off mechanisms, in which stronger survival signaling may favor cancer risk while reducing neurodegenerative vulnerability, whereas weaker survival signaling may reduce cancer risk while increasing susceptibility to neurodegeneration. In the figure, ↑ indicates increased or upregulated pathway activity, whereas ↓ indicates decreased or downregulated pathway activity. Red denotes cancer/oncogenesis-associated pathways or outcomes, and blue denotes neurodegeneration-associated pathways or outcomes. Connecting lines indicate shared upstream influences or proposed pathway relationships; they are intended to summarize conceptual relationships rather than imply a single linear causal sequence.

Both neurodegenerative disorders such as Alzheimer’s disease (AD) and malignant neoplasms such as carcinomas of the breast are major public health challenges for the aging populations in the US and worldwide [1,2,3]. While oncogenesis involves dysregulated cell-cycle re-entry, unchecked proliferation, de-differentiation, resistance to apoptosis, and metabolic reprogramming [4], in neurodegeneration, there is progressive synaptic failure [5], neuronal loss, neuroinflammation, and impaired proteostasis with the accumulation of aggregated proteins [6] (Figure 1). In general terms, these diseases seem distinct, but evidence increasingly suggests they occupy opposite ends of a shared biological spectrum [7]. Over the past decade, population and experimental studies have consistently reported an inverse relationship between cancer and AD, wherein a history of cancer is linked to a reduced risk of developing AD, and conversely, AD is associated with a lower incidence of many malignancies [7,8].

**Figure 2:**
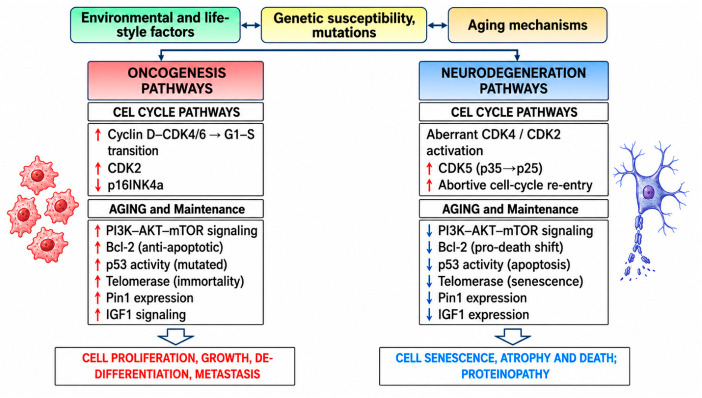
**Opposing molecular pathways in oncogenesis and neurodegeneration.** Common environmental, genetic, and aging-related determinants influence signaling pathways that regulate cell-cycle control, survival, proteostasis, and cellular maintenance in opposite directions in cancer and neurodegenerative disorders. In cancer, activation of Cyclin D–CDK4/6 and CDK2 pathways, reduced p16^INK4a activity, increased PI3K–AKT–mTOR signaling, increased BCL-2-mediated survival signaling, altered or mutant p53 pathway function, increased telomerase activity, increased PIN1 expression, and increased IGF1 signaling favor proliferation, growth, dedifferentiation, resistance to apoptosis, and metastasis. In neurodegenerative disorders, aberrant cell-cycle re-entry, CDK5 dysregulation, reduced PI3K–AKT–mTOR signaling, reduced BCL-2 survival signaling, dysregulated p53-associated apoptotic signaling, reduced telomerase activity, reduced PIN1 expression, and impaired IGF1 signaling favor cellular senescence, neuronal atrophy, cell death, and proteinopathy. This opposing regulation provides a mechanistic framework in which oncogenesis and neurodegeneration represent divergent outcomes of shared aging-related signaling pathways. In the figure, ↑ indicates increased or upregulated pathway activity, whereas ↓ indicates decreased or downregulated pathway activity. Red denotes cancer/oncogenesis-associated pathways or outcomes, and blue denotes neurodegeneration-associated pathways or outcomes. Connecting lines indicate shared upstream influences or proposed pathway relationships; they are intended to summarize conceptual relationships rather than imply a single linear causal sequence.

Mechanistically, this paradox may reflect opposing regulation of core cellular pathways dysregulated during aging that modulate cell survival, stress responses, and energy metabolism [9]. Pathways that promote cell survival and proliferation in cancer, such as phosphoinositide 3-kinase/protein kinase B (PI3K/Akt), mechanist target of rapamycin (mTOR) signaling, altered p53 function, enhanced DNA damage tolerance, and shifts toward anabolic metabolism, are often downregulated or dysfunctional in AD, where neurons exhibit heightened vulnerability to stress, impaired autophagy, mitochondrial dysfunction, and activation of pro-apoptotic cascades [10,11,12,13,14] (Figure 2). Conversely, mechanisms that constrain proliferation or promote cell-cycle arrest and apoptosis may protect against cancer but increase susceptibility to neurodegeneration in post-mitotic neurons [10] (Figure 2). Furthermore, dysregulation of aging-related mechanisms such as cell senescence, proteostasis and immunoregulation might have opposing effects in carcinogenesis and neurodegeneration [15,16,17] (Figure 1).

The interpretation of this inverse relationship is complicated by the impact of cancer therapeutics on the nervous system. Some anticancer agents can exacerbate cognitive dysfunction by disrupting neurogenesis, synaptic plasticity, or mitochondrial function [18,19], whereas others, particularly targeted therapies and immunomodulatory agents, may intersect with pathways implicated in AD pathogenesis, including inflammation, protein aggregation, and cellular senescence, and might be neuroprotective. This issue has become increasingly relevant as precision oncology expands, targeting oncogenic signaling networks. Moreover, some anticancer therapies show potential for repurposing in AD treatment [20]. Elucidating how these interventions influence aging-associated and neurodegenerative pathways may not only clarify the cancer–AD inverse correlation but also reveal novel therapeutic targets that bridge oncology and neurodegeneration.

The aims of this review are fourfold: (**i**) to further clarify the relationships between specific types of cancers and neurodegeneration, (**ii**) to use this understanding as a foundation for exploring the mechanistic links between selected malignant neoplasms and neurodegenerative disorders, (**iii**) to contextualize these relationships within shared and distinct aging pathways (e.g., cell senescence, proteostasis) and (**iv**), based on this, examine emerging cancer therapeutics that might be relevant to AD and related dementias and vice versa.

This narrative review was conducted using a targeted literature search to identify key epidemiological, mechanistic, and translational studies examining the relationship between cancer and neurodegenerative disorders. Publicly available databases, including PubMed/MEDLINE, Web of Science, and Google Scholar, were searched for relevant articles published approximately between 2000 and 2025, with emphasis on recent studies from the past decade. Search terms included combinations of “Alzheimer’s disease,” “cancer,” “neurodegeneration,” “inverse association,” “epidemiology,” “cell cycle,” “senescence,” and “proteostasis”. Studies were selected based on relevance to the central themes of the review, prioritizing large epidemiological cohort studies, meta-analyses, and mechanistic investigations with direct implications for cancer–neurodegeneration relationships. Additional articles were identified through reference lists of key publications. Given the narrative nature of this review, formal systematic inclusion/exclusion criteria were not applied; however, efforts were made to include representative and high-quality studies across different methodological approaches to minimize selection bias.

By integrating insights from recent studies, this review seeks to identify unifying biological principles and translational opportunities that may inform novel therapeutic strategies at the intersection of oncology, neurodegeneration, and aging.

### 1.1. Inverse Associations Between Alzheimer’s Disease and Specific Malignant Neoplasms

Aging is the most common shared risk factor both for malignant neoplasms and neurodegenerative disorders [21,22]. Roughly 6 in 10 new cancer cases in the US occur in people 65 or older, and, among them, the most common are prostate (most common cancer in men), breast (most common in women), lung (both sexes), colon–rectal (both sexes), bladder (older men), pancreatic, melanoma (more in men) and non-Hodgkin lymphoma [23]. Most cases of newly diagnosed advanced cognitive impairment such as dementia (9 out of every 10) in the US occur in people aged 65 or older. Alzheimer’s disease is the most common cause of dementia and the most common neurodegenerative disorder of the aging population, affecting over 6 million in the US [24], followed by Lewy body disease (LBD), fronto-temporal lobar dementia (FTLD), vascular dementia (VaD) and limbic-predominant age-related TDP-43 encephalopathy [1,25,26].

In AD, amyloid beta protein (Aβ) and tau accumulate [24], causing gradual degeneration of neural networks such as the Default Mode Network (DMN), which includes the hippocampus, posterior cingulate, and medial temporal or prefrontal regions and other intrinsic brain networks [27,28]. This damage to neurons and their connections results in declining memory, reasoning, and social abilities. These networks degenerate in a predictable pattern, starting with hub regions crucial for memory (hippocampus, entorhinal cortex) and spreading to language, reasoning, and social areas [29]. It is increasingly recognized that AD commonly presents as a mixed pathology, with over 50% of cases exhibiting co-occurring α-synuclein pathology (Lewy bodies), vascular changes, and TDP-43 aggregates [30,31].

Multiple observational studies, meta-analyses, and Mendelian randomization investigations support an inverse relationship between cancer and AD [32,33,34,35]. However, many studies rely on clinical diagnoses rather than neuropathological confirmation, and the term “AD” is frequently used to describe a broader syndrome of cognitive impairment or dementia (Table 1) [36]. Neuropathological studies demonstrate that a substantial proportion of such cases harbor non-AD or mixed pathologies, including LBD and VaD [30,31] (Table 2). This distinction is critical, as elucidating the biological relationship between neurodegenerative disease and oncogenesis requires pathology-resolved analyses to determine whether specific neurodegenerative disorders are differentially associated with specific cancer types.

For example, one meta-analysis found that AD patients had a roughly 40% reduced risk of cancer (relative risk 0.60), and a prior cancer history was associated with a 15% reduced risk of AD (RR 0.85) [37] (Table 1). This study included 18,887 participants to calculate cancer risk among AD patients and 11 studies, with a total of 5,607,076 participants (Table 1). This study showed that the strongest inverse relationship was with smoking-related cancers (lung, head/neck, bladder, esophagus), with less consistent findings for hormonally driven cancers (e.g., breast, prostate) (Table 2). The age range of the cancer cases was between 65 and 75 y/o and, for the AD individuals, between 65 and 85 y/o. The study emphasized the need for longitudinal studies, with more specific designs including biomarker or neuropathology-confirmed AD and cancer subtype-specific analyses.

A more recent review and meta-analysis of 19 cohort and 3 case–control studies, involving over 9.6 million individuals, reported that cancer was associated with decreased incidence of AD (hazard ratio ≈ 0.89; 95% CI, 0.79–1.00) in cohort studies and an odds ratio ≈ 0.75 (95% CI, 0.61–0.93) in case–control studies [38] (Table 1). The meta-analysis itself did not report detailed effect estimates by each individual cancer type in the main pooled results. However, closer analysis of the data by cohorts suggests that non-melanoma skin cancer tends to show one of the more consistent inverse associations with AD, presumably because it has low mortality and less survival bias (Table 2). Prostate and colorectal cancers also trend inversely but show more variability across studies (Table 2). For breast cancer, only one cohort was included, suggesting an inverse but modest association (Table 2). The authors concluded that the modest inverse correlation between cancer and AD may reflect shared inverse etiological mechanisms but is not likely related to diagnostic bias, competing-risk bias, or insufficient or inappropriate control for confounding factors [38]. Additional retrospective data indicate that among dementia patients with a prior history of cancer, cognitive baseline scores were higher and decline slower compared with dementia patients without cancer history [39]. More recent studies have used Mendelian randomization to provide evidence of causality. Genetic variants associated with increased risk of cancer were associated with a reduced risk of AD [40]. They found that genetically predicted lung and breast cancers (OR) were associated with 9.0% and 5.9% lower odds of AD, respectively. Genetically predicted smoking-related cancers showed a more robust inverse association with AD than non-smoking-related cancers (OR 0.95, 95% CI 0.92–0.98, *p* = 0.0026, vs. OR 0.98, 95% CI 0.97–0.995, *p* = 0.0091) (Table 1). These results support the possibility that the association is not purely due to chance or simple confounding. Yet, it is important to consider these results with caution as, while this inverse relationship is consistent [41], it is not universal. Some studies show weaker associations or null results, and variation exists by cancer type, time since diagnosis, age, and survival bias [38,42,43].

Conversely, a lower malignant neoplasm incidence has been reported in AD populations. A clinical study conducted in China indicated that patients with AD demonstrated a reduced overall risk of cancer (standardized incidence ratio approximately 0.88) compared to the general population, with notably lower lung cancer rates observed among male patients [44] (Table 1). Specifically, patients with AD were protected from lung cancers (SIR = 0.75), and in subgroup analyses, women, patients aged 60–79 years, and those diagnosed as having AD for more than 1 year were more likely to be protected from cancers. A similar study in a South Korean cohort observed a significantly lower likelihood of overall malignancy (HR 0.63) among AD patients compared with controls [45]. This protective effect against certain organ-specific cancers persisted over the 16-year follow-up period, except for in kidney cancer and hematologic malignancies [45]. A meta-analysis across many cohorts reported that people with AD are epidemiologically less likely to develop cancer than individuals without AD (relative risk 0.53), suggesting the inverse relationship is robust across settings [8]. In sub-analyses for site-specific cancers, only lung cancer showed a significantly decreased risk (RR 0.72; 95% CI 0.56–0.91) [46].

Important methodological limitations remain, including survival and competing-risk bias, differences in medical surveillance and diagnostic ascertainment, heterogeneity in cancer treatments, and variability in follow-up duration [47,48]. Cancer patients may die earlier or undergo different patterns of clinical monitoring, potentially influencing the likelihood of subsequent dementia diagnosis. Moreover, the biological mechanisms underlying these associations remain incompletely understood, and current evidence does not establish a direct causal protective effect of cancer against AD. Thus, while the inverse cancer–AD relationship is significant and increasingly supported by population-level studies, it should be interpreted as a complex and potentially heterogeneous association rather than a universal or definitive biological rule.

**Table 1:** **Studies** **examining cancer–Alzheimer’s disease associations.**

Ref.	Study/Source	Design/Data Source	Sample/Population	Main Finding	Effect Size/95% CI
[36]	Roe et al., Neurology (2010)	Prospective cohort; CHS-Cognition Substudy	n = 3020; age ≥ 65	Prevalent cancer was associated with lower AD risk; the association was stronger for pure AD than for any AD.	Any AD: HR 0.72, 95% CI 0.52–0.997; pure AD: HR 0.57, 95% CI 0.36–0.90.
[41]	Driver et al., BMJ (2012)	Prospective cohort; Framingham Heart Study	n = 1278; 9164 person-years	Cancer survivors had lower risk of probable AD; participants with probable AD had lower subsequent cancer risk.	Cancer survivors: probable AD HR 0.67, 95% CI 0.47–0.97; smoking-related cancers HR 0.26, 95% CI 0.08–0.82; subsequent cancer after probable AD HR 0.39, 95% CI 0.26–0.58.
[32]	Musicco et al., Neurology (2013)	Population-based incidence study; Northern Italy registry data	>1 million residents; 2004–2009 observation period	Bidirectional inverse association: AD dementia patients had reduced cancer incidence and cancer patients had reduced AD dementia incidence.	Abstract reports cancer risk in AD dementia was approximately halved, and AD dementia risk in cancer patients was reduced by approximately 35%.
[44]	Ou et al., Neuroepidemiology (2013)	Nationwide population-based study; Taiwan NHIRD	6960 AD patients	AD patients had reduced overall cancer incidence, with lower lung cancer rates particularly noted.	Overall cancer: SIR 0.88, 95% CI 0.80–0.97; lung cancer: SIR 0.75, 95% CI 0.57–0.98.
[37]	Papageorgakopoulos et al., Hell J Nucl Med (2017)	Systematic review and meta-analysis	18,887 participants for cancer risk among AD patients; 11 studies totaling 5,607,076 participants for AD risk among cancer patients	Meta-analysis supported an inverse association in both directions.	AD patients had reduced cancer risk: RR 0.60, 95% CI 0.45–0.79; prior cancer history associated with reduced AD risk: RR 0.85, 95% CI 0.81–0.88.
[38]	Ospina-Romero et al., JAMA Network Open (2020)	Systematic review and meta-analysis with bias evaluation	22 studies; 9,630,435 individuals	Cancer was associated with decreased AD incidence; diagnostic bias and competing-risk bias were unlikely to fully explain the association.	Cohort studies: HR 0.89, 95% CI 0.79–1.00; case-control studies: OR 0.75, 95% CI 0.61–0.93.
[40]	Seddighi et al., Scientific Reports (2019)	Mendelian randomization analysis	Cancer and AD GWAS datasets	Genetically predicted cancer liability was associated with reduced odds of AD for selected cancers.	Lung cancer: OR 0.91, 95% CI 0.84–0.99; breast cancer: OR 0.94, 95% CI 0.89–0.99; leukemia: OR 0.98, 95% CI 0.96–0.995; smoking-related cancers: OR 0.95, 95% CI 0.92–0.98; non-smoking-related cancers: OR 0.98, 95% CI 0.97–0.995.
[45]	Kang et al., Cancers (Basel) (2023)	Large matched Korean cohort; longitudinal follow-up	24,664 AD patients and 98,656 matched controls	AD was associated with lower overall malignancy and several site-specific cancers during 16-year follow-up.	Overall cancer: adjusted HR 0.63, 95% CI 0.59–0.68. Stronger site-specific inverse associations included pancreatic HR 0.50, lung HR 0.64, colorectal HR 0.67, and hepatic HR 0.60.
[8]	Zheng et al., Behavioural Brain Research (2025)	Epidemiological meta-analysis plus transcriptomic/APOE tumor-immunity analysis	Published epidemiological datasets; TCGA pan-cancer transcriptomic analysis	Recent meta-analysis supported a bidirectional inverse epidemiological relationship and proposed APOE/tumor-immunity links.	AD patients were less likely to develop cancer: RR 0.53; cancer patients were less likely to develop AD: RR 0.61.
[35]	Dong et al., Journal of Translational Medicine (2023)	Two-sample Mendelian randomization, two-step MR, colocalization, and TCGA transcriptomic analysis	42,034 AD patients and 609,951 cancer patients from GWAS Catalog-derived datasets; 33 TCGA cancer types for transcriptomic analyses	Increased genetic AD risk showed a significant causal influence on reduced cancer risk; VLDL and PVRIG were proposed as molecular links.	No single overall HR/OR is appropriate for this summary table; the study reported significant negative causal effects of AD risk on several cancer outcomes and no significant reverse cancer-to-AD causal effect.

Abbreviations: AD, Alzheimer disease; CHS, Cardiovascular Health Study; CI, confidence interval; GWAS, genome-wide association study; HR, hazard ratio; MR, Mendelian randomization; NHIRD, National Health Insurance Research Database; OR, odds ratio; RR, relative risk; SIR, standardized incidence ratio; TCGA, The Cancer Genome Atlas; VLDL, very-low-density lipoprotein. Effect sizes and confidence intervals are reported as presented in the original studies or in the revised manuscript text whenever available. When a study did not report one global summary estimate appropriate for this table, the entry summarizes the principal direction and key analytical result.

### 1.2. Inverse and Disease-Specific Cancer Associations in Neurodegenerative Disorders Beyond AD

Although most population-based studies have focused on the relationship between AD and cancer, other neurodegenerative disorders such as LBD, VaD, and FTLD often overlap clinically and neuropathologically with AD or present with similar cognitive impairment [30,31]. Determining whether the inverse association with cancer extends beyond AD may help identify shared versus disorder-specific mechanisms linking neurodegeneration and malignancy (Table 2).

Lewy body disease comprises a heterogeneous spectrum of disorders that includes dementia with Lewy bodies (DLB), Parkinson’s disease dementia (PDD), and idiopathic Parkinson’s disease (iPD) [49,50]. These conditions are characterized by progressive α-synuclein aggregation and dissemination involving subcortical, limbic, and neocortical regions [51]. Genetic risk factors include variants in α-synuclein (SNCA), glucosylceramidase beta 1 (GBA), leucine-rich repeat kinase 2 (LRRK2), apolipoprotein E (APOE), and transmembrane protein 175 (TMEM175), which influence α-synuclein aggregation, lysosomal function, and neuroinflammatory responses [52,53,54]. More than 30% of LBD cases also show mixed pathology, including coexisting amyloid-β, tau, TDP-43, and cerebrovascular lesions [55].

Although evidence remains limited, case–control and cohort studies reported that individuals with DLB were less likely to have a prior history of cancer compared with cognitively normal controls (OR ≈ 0.44), suggesting a potential inverse association, although cancer subtypes were not specified [56]. A more recent study using neuropathologically confirmed cases from the National Alzheimer’s Coordinating Center (NACC) showed evidence that prevalent and incident cancer diagnoses were associated with better global cognition (BF = 26). Moreover, cancer diagnosis was inversely associated with TAR DNA-binding protein 43 (TDP-43) pathology and showed weaker evidence for lower Lewy body pathology (BF = 3.2) [57,58]. Other studies reported inconsistent associations between cancer history and Lewy body pathology, while no clear association was observed with TDP-43 burden [50,51]. However, these studies did not specify cancer subtypes.

The inverse association between cancer and PD appears considerably stronger. A large systematic review and meta-analysis including nearly 18 million participants found that individuals with PD had approximately 15% lower overall cancer risk, while individuals with cancer had approximately 26% lower risk of subsequently developing PD [58,59] (Table 2). Earlier meta-analyses similarly reported overall cancer risk reductions ranging from 17% to 27% in PD populations [59]. Importantly, the relationship appears highly cancer-type-specific. Large cohort studies and meta-analyses consistently report decreased risks of smoking-related cancers, lung carcinoma, prostate cancer, bladder cancer, and several gastrointestinal malignancies in PD populations compared with age-matched controls [60,61,62] (Table 2).

In contrast, melanoma and certain brain tumors show significantly increased risk in PD, with melanoma representing one of the most reproducible positive associations across studies [62,63] (Table 2). Associations with breast, thyroid, and hematological malignancies remain inconsistent across studies [63]. Collectively, these findings suggest that PD–cancer associations likely reflect cancer-specific interactions involving cell-cycle regulation, apoptosis, and stress response pathways.

As for FTLD, this comprises a clinically and molecularly heterogeneous group of disorders characterized by selective degeneration of the frontal and temporal lobes [64,65]. The most common genetic causes involve mutations in microtubule-associated protein tau (MAPT), progranulin (GRN), and C9orf72, with additional contributions from TANK-binding kinase 1 (TBK1), valosin-containing protein (VCP), and charged multivesicular body protein 2B (CHMP2B) [66,67]. These mutations are associated with abnormal aggregation of tau, TDP-43, or fused in sarcoma (FUS) proteins [66,67]. Several of these pathways have also been implicated in oncogenic regulation [68,69]. Only a limited number of studies have examined cancer prevalence in FTLD. A Finnish cohort reported significantly lower cancer prevalence in FTLD patients compared with AD patients and cognitively normal controls [70]. Cancer prevalence in FTLD was 9.7%, compared with 18.7% in AD and 17.4% in cognitively normal controls (Table 2). This reduced prevalence was observed regardless of C9orf72 carrier status, suggesting a potentially stronger inverse association than that observed in AD. Interestingly, C9orf72 repeat expansions have also been associated with increased melanoma risk, indicating that specific FTLD genetic subgroups may show distinct cancer susceptibilities despite the overall inverse trend [70]. Further multicenter studies are needed to validate these findings and determine whether specific FTLD subgroups display distinct cancer-risk profiles.

Collectively, these findings support the concept that opposing regulation of fundamental aging-related pathways, including cell-cycle control, DNA damage responses, proteostasis, mitochondrial function, senescence, and apoptosis, may differentially predispose individuals to specific neurodegenerative disorders.

**Table 2:** **Association** **between specific types of cancers and neurodegenerative disorders.**

Cancer Type	AD	PD	LBD/DLB	FTLD	Mechanistic Interpretation	Key References (Current Revised Manuscript Numbering)
Lung	↓↓	↓↓	—	↓?/—	Most consistent inverse evidence among smoking-related cancers in AD and PD. Direct DLB/LBD and FTLD cancer-subtype data are sparse; FTLD support is mainly for lower overall cancer prevalence, not lung-specific risk. Potential contributors include smoking/survival/competing-risk effects, p53/DNA damage response/autophagy programs, immune tone, and treatment/ascertainment effects.	AD: [32,37,38,40,44,46]; PD: [58,60,61,62,63]; LBD/DLB overall: [56,57]; FTLD overall: [70]; Mechanisms/limits: [9,10,47,48,71,72,73]
Prostate	↔/↓	↔/↓	—	↓?/—	Evidence is heterogeneous. AD evidence comes from cancer-type subgroup analyses and cohort data, but the direction is less consistent than for smoking-related cancers. PD meta-analyses report mixed/reduced risk depending on study design and population. Mechanistic considerations include androgen receptor-PI3K crosstalk, IGF/insulin signaling, and treatment-related confounding such as androgen deprivation therapy.	AD: [37,38,45]; PD: [58,60,61,63]; FTLD overall: [70]; Mechanisms: [74,75,76,77]
Colorectal	↔/↓	↓↓	—	—	AD evidence is compatible with an inverse overall cancer-AD association, but CRC-specific evidence is less consistently singled out. PD shows more consistent reduced colorectal/colon/rectal cancer risk in meta-analyses. Mechanistic links include WNT/beta-catenin and cell-cycle signaling, inflammation, and gut-brain axis effects; recent experimental evidence links AD-like pathology, microbiota, and inflammatory tolerance to reduced colorectal tumorigenesis.	AD: [37,38,45]; PD: [58,60,61,63]; Mechanisms: [78,79,80]
Breast	↓	↔/↓	—	—	AD associations are generally inverse but modest/variable. PD evidence is mixed and may depend on sex, genetics, therapy, and surveillance. Mechanistic considerations include IGF/insulin, PI3K-AKT/MAPK signaling, endocrine influences, and treatment-related confounding.	AD: [37,38,40,45]; PD: [58,60,61,63]; Mechanisms: [74,75,76]
Non-melanoma skin	↓↓	↔/↓	—	—	Non-melanoma skin cancer is one of the cancer types most often reported as inversely associated with AD, partly because lower mortality may reduce competing-risk bias. In PD, non-melanoma skin cancer is generally null or modestly reduced, in contrast to melanoma. Mechanistic considerations include immune surveillance, UV-DNA damage responses, p53, and senescence pathways.	AD: [38]; PD: [58,60,61,63]; Mechanisms/limits: [10,47,48,81]
Melanoma	↔	↑↑	—	↑?/—	Melanoma is the major exception to the generally inverse PD-cancer pattern and shows a reproducible positive association with PD. Direct DLB/LBD melanoma-specific evidence is sparse. FTLD evidence is limited to C9ORF72 repeat-expansion carriers and should not be generalized to all FTLD. Proposed biology includes melanin/dopamine-related pathways, mitochondrial stress, alpha-synuclein/immune mechanisms, genetics, and surveillance effects.	PD: [58,60,61,62,63]; C9ORF72/FTLD subgroup: [69]; Mechanisms/background: [51,53,54,82,83]
Hematologic	↔/↓	↔/↓	—	—	Associations are heterogeneous by subtype and treatment. AD cohort data do not consistently support the same inverse relationship as for lung or non-melanoma skin cancer. PD analyses suggest reduced or mixed risks depending on subtype and genetic background. Mechanistic considerations include cytokine milieu, immune aging, clonal hematopoiesis, inflammatory tone, and therapy effects.	AD: [45]; PD: [58,60,61,63]; Mechanisms: [17,81,83,84]

References are shown using the current numbering in the revised manuscript. The arrows summarize the direction and consistency of the reported epidemiological association, not causality. Symbol key: ↓↓, relatively consistent inverse association; ↓, inverse association; ↔/↓, mixed or weak evidence with possible inverse trend; ↔, no consistent association; ↑, positive association; ↑↑, relatively consistent positive association; —, insufficient or not reported; ?, limited/indirect evidence requiring cautious interpretation. Abbreviations: AD, Alzheimer’s disease; PD, Parkinson’s disease; LBD, Lewy body disease; DLB, dementia with Lewy bodies; FTLD, frontotemporal lobar degeneration; CRC, colorectal cancer; ADT, androgen deprivation therapy; AR, androgen receptor; PI3K, phosphoinositide 3-kinase; AKT, protein kinase B; MAPK, mitogen-activated protein kinase; mTOR, mechanistic target of rapamycin; IGF, insulin-like growth factor; DNA, deoxyribonucleic acid; C9ORF72, chromosome 9 open reading frame 72; TREM2, triggering receptor expressed on myeloid cells 2. Cross-check note: The LBD/DLB column is intentionally conservative because the revised manuscript cites overall DLB/LBD cancer or neuropathology associations but does not provide cancer-subtype-specific DLB/LBD estimates. The FTLD entries are also conservative because current evidence primarily supports lower overall cancer prevalence in FTLD, with melanoma evidence limited to C9ORF72 repeat-expansion carriers.

### 1.3. Shared and Divergent Aging Mechanisms Linking Cancer and Neurodegeneration

#### 1.3.1. Diverging Cellular Outcomes Despite a Shared Risk Landscape for Cancer and Neurodegeneration

Neurodegenerative disorders and malignant neoplasms share several major risk factors, including advanced age, lifestyle-associated exposures, and metabolic syndromes such as obesity and diabetes (Figure 1). In addition, certain genetic variants influence susceptibility to both disease classes, often in opposing directions. These conditions are further unified by core aging-related cellular stressors, including oxidative damage, mitochondrial dysfunction, impaired proteostasis, genomic instability, and chronic inflammation [4,85,86,87] (Figure 1). Given this shared risk panorama, the inverse association between malignant transformation and neurodegeneration raises a central biological question. One central hypothesis is that while aging-related stresses broadly increase vulnerability, downstream cellular responses diverge along distinct molecular trajectories (Figure 1) [86]. In some individuals, stress response pathways preferentially activate pro-survival and proliferative signaling such as PI3K–AKT–mTOR activation, suppression of p53-mediated apoptosis, and MYC-driven cell-cycle entry, thereby increasing cancer risk [4,88] (Figure 1). In others, the same stresses may instead engage pathways favoring damage sensing, growth arrest, and cell loss, including p53–p21 signaling, forkhead box O (FOXO)-dependent stress responses, mitochondrial apoptosis, and senescence programs, predisposing them to neurodegeneration [89,90] (Figure 1). These divergent outcomes may reflect differences in regulatory “set points” regulating cell-cycle control, proteostasis, and metabolic adaptation (Figure 1) [35,91].

This concept is supported by transcriptomic analysis studies that examined aging-associated gene expression changes across tissues and vertebrate models and found that aging trajectories aligned more closely with degenerative disease signatures, while moving in the opposite direction from cancer-associated transcriptional programs [91]. Importantly, the study also identified antagonistic effects at the genomic level, in which many shared risk alleles showed opposite directions of association for cancer versus chronic degenerative disorders [91]. These findings support the idea that cancer and neurodegeneration may represent divergent transcriptional and genetic trajectories of aging-related stress responses, rather than completely unrelated disease processes.

For example, increased resistance to apoptosis mediated by reduced p53 activity, increased B-cell lymphoma 2 (BCL-2) family signaling, or chronic activation of mTOR may promote oncogenic expansion while simultaneously protecting neurons from acute cell death and synaptic loss, thereby lowering AD risk (Figure 1) [92,93]. Conversely, enhanced p53 activity, impaired mTOR signaling, excessive or dysregulated autophagy, or heightened activation of proteostatic clearance pathways such as the ubiquitin/proteasome system and lysosomal degradation might limit malignant transformation but increase vulnerability to synaptic dysfunction, neuronal loss, and protein aggregation characteristic of AD [94] (Figure 1).

Thus, the inverse relationship between cancer and neurodegeneration may reflect a fundamental trade-off in aging biology, in which pathways that prioritize cellular survival, growth, and metabolic flexibility confer oncogenic risk, whereas pathways emphasizing cellular senescence, damage detection, clearance, and growth suppression protect against cancer at the expense of increased susceptibility to neurodegenerative disease (Figure 1). These proposed mechanisms remain largely hypothetical and are supported primarily by preclinical and associative evidence rather than direct causal demonstration in humans.

#### 1.3.2. Core Signaling Pathways Promoting Cell Survival vs. Mortality in Cancer and Neurodegeneration

Consequently, multiple mechanistic hypotheses have been advanced, most of which focus on the concept that malignant transformation and neurodegeneration are contrasting dysregulations of core cellular processes [71]. The main pathways often cited are dysregulation of *(i) cell cycle, proliferation, and apoptosis; (ii) cellular maintenance, stress response, and aging; and (iii) shared DNA repair mechanisms (Figure 2)* [72,73]. Dysregulation of cell-cycle control represents a fundamental mechanistic divergence between cancer and AD, despite both conditions arising in the context of aging and cellular stress. In cancer, loss of cell-cycle arrest is a defining hallmark, driven by sustained activation of cyclin-dependent kinases (CDKs) that promote uncontrolled proliferation [78]. Hyperactivation of CDK4 and CDK6, often through cyclin D amplification or loss of CDK inhibitors such as p16^INK4a, facilitates G1–S phase transition and bypass of senescence checkpoints [78]. Aberrant activation of CDK2 further supports DNA replication in the presence of genomic instability, while inactivation of p53 and RB signaling removes critical restraints on CDK activity, enabling malignant transformation [4] (Figure 2).

In contrast, neurons in AD are terminally differentiated and normally maintain permanent cell-cycle arrest. Paradoxically, AD brains exhibit evidence of abortive cell-cycle re-entry, marked by inappropriate activation of CDK4, CDK2, and CDK5 in vulnerable neuronal populations [95]. Unlike cancer, this aberrant CDK activation does not lead to proliferation but instead triggers neuronal stress and cell death. Pathological deregulation of CDK5, driven by cleavage of its activator p35 to p25, results in sustained kinase activity that promotes tau hyperphosphorylation, synaptic dysfunction, and neurodegeneration [96] (Figure 2). Thus, while cancer reflects failure of cell-cycle arrest through CDK-driven proliferation, AD represents a lethal attempt at cell-cycle re-entry in post-mitotic neurons, culminating in neurodegeneration rather than division (Figure 2).

**Figure 3:**
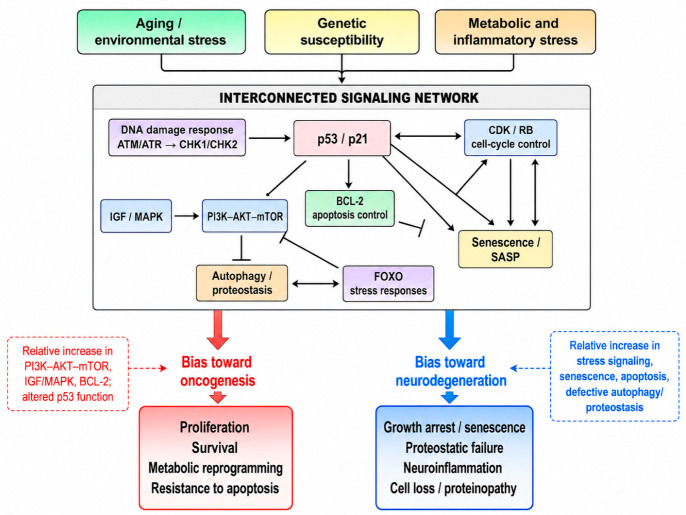
**Interconnected signaling network linking oncogenesis and neurodegeneration.** Aging and environmental stress, genetic susceptibility, and metabolic or inflammatory stress converge on an interconnected signaling network that includes DNA damage response pathways, p53/p21 signaling, CDK/RB-mediated cell-cycle control, IGF/MAPK signaling, PI3K–AKT–mTOR signaling, BCL-2-mediated apoptosis control, FOXO stress responses, autophagy/proteostasis, and senescence/SASP pathways. These modules do not act independently but interact as a stress response network that can be biased toward different cellular outcomes. Relative activation of PI3K–AKT–mTOR, IGF/MAPK, BCL-2 survival signaling, and altered p53 function may favor oncogenesis by promoting proliferation, survival, metabolic reprogramming, and resistance to apoptosis. In contrast, increased stress signaling, senescence, apoptosis, and defective autophagy/proteostasis may bias vulnerable post-mitotic cells toward neurodegeneration, characterized by growth arrest, proteostatic failure, neuroinflammation, cell loss, and proteinopathy. The figure illustrates how shared signaling pathways may diverge toward malignant transformation or neurodegenerative vulnerability depending on cellular context. In the figure, arrows indicate proposed activating or directional pathway relationships, whereas blunt-ended lines indicate inhibitory or suppressive relationships. Red denotes pathway bias toward oncogenesis, and blue denotes pathway bias toward neurodegeneration. Dashed boxes and dashed arrows indicate relative pathway biases rather than absolute disease-specific changes.

**Figure 4:**
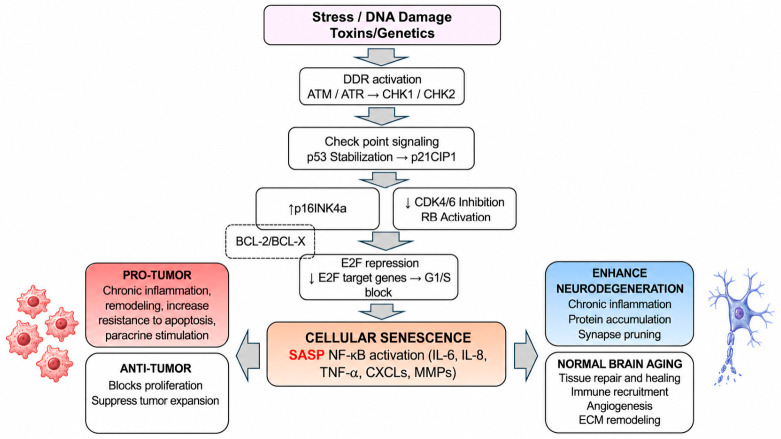
**Cellular senescence as a central outcome of stress-induced DNA damage linking tumorigenesis and neurodegeneration**. Cellular stressors, including DNA damage, toxins, and genetic factors, activate the DNA damage response (DDR) through ATM/ATR signaling and downstream CHK1/CHK2 pathways. This response promotes checkpoint signaling characterized by stabilization of p53 and induction of p21^CIP1, together with activation of p16^INK4a and inhibition of CDK4/6 leading to RB activation. These pathways converge on repression of E2F target genes and blockade of the G1/S cell-cycle transition, resulting in stable growth arrest and establishment of cellular senescence. Senescent cells develop a senescence-associated secretory phenotype (SASP), characterized by activation of NF-κB signaling and secretion of inflammatory mediators including IL-6, IL-8, TNF-α, CXCL chemokines, and matrix metalloproteinases (MMPs). The biological consequences of senescence are context-dependent. In cancer, senescence can exert anti-tumor effects by blocking proliferation and suppressing tumor expansion, but it may also promote pro-tumorigenic effects through chronic inflammation, tissue remodeling, resistance to apoptosis, and paracrine stimulation. In the nervous system, senescence-associated processes may contribute to neurodegeneration through chronic inflammation, protein accumulation, and synaptic alterations, while under physiological conditions senescence-related responses may support normal brain aging, including tissue repair, immune recruitment, angiogenesis, and extracellular matrix remodeling. This model illustrates cellular senescence as a shared stress response mechanism linking aging-related pathways to divergent outcomes in cancer and neurodegenerative disease. In the figure, arrows indicate directional signaling steps within the DNA-damage response and senescence pathway. Red denotes tumor-related outcomes, and blue denotes neurodegeneration-related outcomes.

These pathways are highly interconnected rather than isolated. PI3K–AKT–mTOR and IGF/MAPK signaling promote anabolic growth, protein synthesis, and resistance to apoptosis, whereas p53–p21 and ATM/ATR–CHK1/CHK2 signaling coordinate DNA damage sensing, cell-cycle arrest, senescence, or apoptosis. CDK/RB signaling determines whether stressed cells continue cycling or enter stable growth arrest, while BCL-2 family proteins regulate mitochondrial apoptotic thresholds. FOXO transcription factors, autophagy–lysosomal pathways, and proteostasis networks provide compensatory stress resistance mechanisms, but their chronic dysregulation can contribute to senescence, impaired protein clearance, and inflammatory SASP signaling (Figure 3 and Figure 4). Thus, the same signaling modules can generate divergent biological outcomes depending on cellular context; that is, in proliferative tissues, they may favor malignant survival and expansion, whereas in post-mitotic neurons and glial cells, they may favor growth arrest, proteostatic failure, neuroinflammation, and degeneration (Figure 2).

Several molecular pathways have emerged as potential common nodes underlying the inverse relationship between cancer and AD, notably peptidyl-prolyl cis/trans isomerase NIMA-interacting 1 (PIN1), p53, and insulin/insulin like growth factor-1 (IGF-1) signaling. PIN1 is a phosphorylation-dependent prolyl isomerase that regulates the conformation and function of numerous proteins involved in cell-cycle progression, transcriptional control, and proteostasis. In cancer, PIN1 is frequently overexpressed and promotes proliferation and tumor progression by stabilizing oncogenic substrates such as cyclin D1, β-catenin, and MYC [97]. In contrast, in AD, PIN1 activity is reduced or functionally impaired, limiting its ability to isomerize phosphorylated tau and thereby favoring tau hyperphosphorylation, aggregation, and neurofibrillary tangle formation [97,98,99,100]. These opposing effects position PIN1 as a key molecular determinant linking oncogenic signaling to tau pathology (Figure 2).

Other shared pathways show similar context-dependent divergence (Figure 1). For instance, in oncogenesis, pathways such as PI3K/AKT/mTOR are often hyperactive (promoting cell growth/survival); in AD, these may be impaired [101]. Similarly, Bcl-2, a pro-survival (anti-apoptotic) protein often upregulated in cancers, is downregulated in AD [101]. Telomerase activity helps cancer cells evade senescence, while in AD, accelerated neuronal death and failure of maintenance machinery contribute to degeneration [102] (Figure 1 and Figure 2). The tumor suppressor p53 represents another critical axis of divergence. In cancer, p53 is commonly mutated or inactivated, allowing damaged cells to evade apoptosis and senescence [9]. In AD, however, increased p53 activation has been associated with neuronal stress responses, mitochondrial dysfunction, and synaptic impairment, suggesting that heightened tumor-suppressive signaling may exacerbate neurodegeneration [9,89,103]. Insulin/IGF-1 signaling further illustrates this inverse regulation. IGF-1 promotes growth, survival, and metabolic activity and is frequently upregulated in cancer, supporting proliferation and resistance to apoptosis [74,75]. In AD, by contrast, IGF-1 signaling is often reduced or dysregulated, contributing to impaired neuronal metabolism, synaptic dysfunction, and increased vulnerability to amyloid-β toxicity [76]. Collectively, differential regulation of PIN1, p53, and insulin/IGF-1 signaling provides a mechanistic framework through which shared aging-related pathways can drive opposing disease outcomes in cancer and AD (Figure 1 and Figure 2).

Although most mechanistic evidence has been developed in relation to AD, several of these pathway relationships are also relevant to PD/LBD and FTLD. In PD and LBD, mitochondrial dysfunction, impaired autophagy–lysosomal clearance, altered kinase signaling, α-synuclein proteostasis, p53-related stress responses, and dysregulated PI3K–AKT–mTOR signaling intersect with pathways that regulate survival, apoptosis, and cellular resilience [10,11]. Molecular mechanisms involving tau, TDP-43, progranulin, C9orf72, TBK1, VCP, inflammatory signaling, and proteostasis pathways may similarly influence the balance between cellular maintenance, degeneration, and oncogenic susceptibility [64,82,104]. Collectively, differential regulation of PI3K–AKT–mTOR, p53, DNA damage signaling, IGF/MAPK, BCL-2, autophagy/proteostasis, and senescence pathways provides a mechanistic framework through which shared aging-related processes can drive opposing disease outcomes in cancer and neurodegenerative disorders (Figure 2 and Figure 3).

#### 1.3.3. Aging Mechanisms Have Opposing Roles in Oncogenesis and Neurodegeneration

Aging is a progressive biological process characterized by the accumulation of molecular and cellular damage, leading to declining tissue resilience and increased vulnerability to disease. The recognized hallmarks of aging include the following [87]: genomic instability, telomere attrition, epigenetic alterations, loss of proteostasis, deregulated nutrient sensing, mitochondrial dysfunction, cellular senescence, stem cell exhaustion, and altered intercellular communication. These interconnected processes collectively shape susceptibility to age-related disorders, including cancer and neurodegeneration. The way in which cells respond to aging-related stress, repair DNA damage, and maintain proteostasis diverges considerably between oncogenesis and neurodegeneration (Figure 2). Cancer cells frequently enhance adaptive stress responses and maintain telomere length (via telomerase or ALT), thereby supporting continued proliferation [95]. Cancer epigenomes are also characterized by global hypomethylation together with gene-specific hypermethylation, promoting malignant transformation and progression [95]. In cancer, survival and maintenance pathways are persistently activated to support growth and stress resistance [78]. In contrast, neurodegenerative disorders are characterized by impaired DNA repair, defective mitochondrial function [105], oxidative stress, reduced proteostatic capacity, and progressive metabolic failure [81] (Figure 3). For example, reactive oxygen species (ROS) are elevated in AD and contribute to neuronal injury, whereas many cancers adapt to oxidative stress by upregulating antioxidant defenses that support survival and proliferation [84,101].

Among the hallmarks of aging, cellular senescence and proteostasis play central roles in determining whether stressed cells undergo proliferation, arrest, clearance, or degeneration [106] (Figure 4). These pathways exert context-dependent and often opposing effects in cancer and neurodegenerative disease. Cellular senescence is a stress-induced state of stable cell-cycle arrest triggered by DNA damage, telomere attrition, oncogene activation, oxidative stress, and mitochondrial dysfunction [107,108]. The senescence program is mediated largely through the p53–p21^CIP1 and p16^INK4a-RB pathways, resulting in cyclin-dependent kinase inhibition, repression of E2F-dependent genes, and chromatin remodeling that stabilizes growth arrest. Senescent cells also develop a senescence-associated secretory phenotype (SASP), characterized by secretion of pro-inflammatory cytokines and chemokines that can initially promote repair but, when persistent, contribute to chronic inflammation and tissue dysfunction [82].

In oncogenesis, senescence functions primarily as a tumor-suppressive mechanism by preventing proliferation of damaged or genomically unstable cells [109]. Cells that fail to properly engage senescence or escape from it are more likely to undergo malignant transformation [109]. In contrast, during brain aging and AD, senescence-like states emerge in both neurons and glial populations, where they exert deleterious effects. Although post-mitotic neurons do not undergo classical replicative senescence, they can develop persistent DNA damage responses and stress-associated senescence-like phenotypes [109]. Astrocytes and microglia can also display canonical senescence markers, including p16^INK4a upregulation, SA-beta-gal activity, and inflammatory secretory signaling [110,111] (Figure 4). Experimental tauopathy studies demonstrated that elimination of senescent glial cells reduces tau pathology, neuroinflammation, and cognitive decline [112]. Similarly, oligodendrocyte progenitor cells near Aβ plaques can acquire senescent-like features, while senolytic therapies alleviate these phenotypes and improve cognition in AD models [113]. Thus, accumulation of senescent cells within the CNS contributes to chronic inflammation, synaptic dysfunction, and impaired neuronal support [114]. Experimental studies in tauopathy mouse models such as PS19 further demonstrated that selective elimination of p16^Ink4a-positive senescent cells using INK-ATTAC reduced inflammation, phosphorylated tau accumulation, neuronal loss, and cognitive decline [112]. Pharmacological senolytic approaches using navitoclax (ABT263) or dasatinib plus quercetin similarly reduced neuroinflammatory and amyloid-related pathology in AD models [112,115], supporting senescence-targeting strategies as potential therapeutics for neurodegenerative disorders.

Proteostasis maintains protein homeostasis through coordinated regulation of protein synthesis, folding, trafficking, and degradation pathways, including autophagy, lysosomal systems, and the ubiquitin–proteasome pathway [116,117,118,119]. Divergent regulation of proteostasis is a major distinguishing feature between cancer and neurodegeneration [116,119]. Malignant cells experience substantial proteotoxic stress due to oncogene-driven growth and elevated protein synthesis [120,121]. To compensate, tumors frequently enhance chaperone systems such as HSP90 and increase proteasome activity to stabilize oncogenic proteins and maintain rapid protein turnover [122,123]. These adaptations support survival under chronic stress conditions and facilitate malignant progression.

In contrast, proteostasis failure is a defining feature of neurodegenerative disorders [104]. Neurons depend on efficient protein quality-control systems to maintain function over decades without cell division, and impairment of proteasomal and autophagy–lysosomal pathways promote accumulation of aggregation-prone proteins, including Aβ, tau, α-synuclein, and TDP-43 [82,124,125,126]. Unlike proliferative cancer cells, post-mitotic neurons cannot dilute toxic proteins through cell division, rendering them highly vulnerable to even modest reductions in proteostatic efficiency [127,128,129,130]. Autophagy illustrates the context-dependent nature of proteostasis regulation. In early tumorigenesis, autophagy suppresses cancer development by removing damaged mitochondria and limiting oxidative stress [131,132,133,134]. However, advanced tumors frequently hijack autophagy to survive metabolic and microenvironmental stress [135,136]. In neurodegenerative disorders, autophagy is often initially activated as a compensatory response but subsequently becomes dysfunctional because of impaired lysosomal clearance and trafficking defects, resulting in stalled autophagic flux and accumulation of autophagic vacuoles [133]. Excessive or defective autophagy may further impair synaptic integrity and contribute to neuronal degeneration [134].

Senescent cells undergo profound proteostatic remodeling characterized by impaired proteasome activity, defective autophagic flux, and accumulation of damaged proteins and organelles [135,136]. In cancer, evasion of senescence preserves proteostatic flexibility and supports continued proliferation under stress [136,137]. In contrast, persistence of senescent glial cells in AD amplifies proteostatic stress through chronic SASP-mediated inflammation and impaired neuronal support [138]. Molecular regulators including mTOR, FOXO transcription factors, and p53 integrate senescence and proteostasis pathways [139,140,141]. Chronic mTOR activation promotes anabolic growth while suppressing autophagy, thereby supporting oncogenesis but impairing protein clearance in AD [142]. Conversely, excessive mTOR inhibition may enhance autophagy and senescence while contributing to synaptic dysfunction and neuronal injury [141,142]. FOXO transcription factors promote stress resistance and autophagy, although sustained FOXO activation may also reinforce growth arrest and neurodegenerative vulnerability [143].

Systemic aging-associated factors, including metabolic dysfunction, impaired immune surveillance, and mitochondrial decline, further influence the balance between senescence and proteostasis [17]. Aging-related reductions in proteostatic capacity increase susceptibility to protein aggregation disorders, while defective immune clearance of senescent cells exacerbates neuroinflammation [87]. At the same time, these processes may constrain unchecked proliferation and partially reduce cancer susceptibility in individuals with neurodegenerative disease [87,136]. Within this framework, the inverse association between malignant neoplasms and neurodegeneration reflects divergent cellular programs for managing chronic genotoxic, metabolic, and proteotoxic stress. These opposing disease trajectories arise from gene–environment interactions that bias cells toward either sustained survival and repair or toward senescence and degeneration [144] (Figure 4). An interesting example is the APOE locus: APOE2 is well established as protective against AD [145], whereas its reported association with increased risk or progression of selected malignancies is less consistent and appears to depend on cancer type, population, and study design [146]. Conversely, APOE4 strongly increases AD risk [147,148,149], but its reported association with reduced incidence or slower progression of some cancers remains heterogeneous and should be interpreted cautiously [150,151,152]. Similar antagonistic effects have been suggested for genes regulating cell-cycle control, DNA repair, telomere maintenance, and growth-factor signaling pathways, including TP53, CDKN2A, ATM, TERT, and IGF-1/PI3K-AKT [153,154,155,156,157,158,159]. Collectively, these findings support a model in which cellular programs favoring survival and repair protect against neurodegeneration at the cost of increased oncogenic potential, whereas pro-degenerative programs constrain malignant transformation while increasing vulnerability to neurodegeneration.

Thus far, this review has highlighted how core signaling pathways and aging-related mechanisms diverge between cancer and neurodegenerative disorders. These differences likely reflect underlying gene–environment interactions that shape cellular stress responses prior to disease onset. Non-cell-autonomous mechanisms such as those driven by paracrine mechanisms involving secreted molecules might also play a role. Many tumors secrete neurotrophic factors, growth factors, and chaperone-like molecules that activate pro-survival pathways in neurons and glial cells (e.g., PI3K–AKT, MAPK–ERK, mTOR), enhancing proteostasis and resistance to stress [77,160]. Conversely, injured neurons and activated glial cells release inflammatory and stress-associated signals that can suppress proliferation and promote senescence or apoptosis in peripheral tissues [16,79,161,162,163]. This bidirectional model is context-dependent; for example, malignancies secrete overlapping repertoires of growth factors, such as IGF-1, VEGF, BDNF, TGF-β, and FGFs [164], which promote neuronal survival and proteostatic pathways relevant to AD, LBD, and FTLD [165]. Tumor cells may also secrete chaperones and related molecules that interact with amyloid aggregates. For example, peripheral tumors can influence AD pathology via secretion of cystatin-C [80], which crosses the blood–brain barrier and enhances microglial amyloid clearance via TREM2 signaling. In experimental models, tumor-bearing mice show reduced Aβ burden and improved cognitive performance, effects dependent on TREM2 and cystatin-C signaling [80,83]. Conversely, neurodegeneration is associated with a systemic stress and inflammatory secretory phenotype that suppresses proliferation and promotes senescence or apoptosis [84,166]. Collectively, these data support a bidirectional model in which neurodegeneration generates a systemic stress and inflammatory milieu. These concepts should be interpreted as hypothesis-generating biological models that require further experimental and clinical validation. Thus, cancer-associated trophic signaling theoretically may confer neuroprotection, whereas neurodegeneration may reciprocally reduce cancer risk by promoting senescence, apoptosis, and immune-mediated tumor suppression.

### 1.4. Translational and Therapeutic Implications

Over the past decade, both neurodegenerative disease research and oncology have undergone extraordinary therapeutic advances, particularly through immunomodulatory and precision-medicine approaches [20]. These advances have generated targeted agents that modulate cell survival, stress responses, epigenetic regulation, inflammatory signaling, and immune pathways [167,168]. Some of these mechanisms are also central to neuronal vulnerability and resilience in AD and related dementias, supporting cancer-to-neurodegeneration drug-repurposing strategies [169,170].

Several tyrosine kinase inhibitors (TKIs), including nilotinib, originally approved for chronic myelogenous leukemia (CML), also inhibit c-Abl, a kinase reported to be aberrantly activated in AD and PD brains [171,172]. Mechanistically, this strategy is particularly relevant to PD and Lewy body dementias, where α-synuclein accumulation and autophagy–lysosomal dysfunction are central pathogenic features [173,174]. In experimental models, nilotinib and bosutinib enhanced autophagy/lysosomal flux, promoted clearance of aggregation-prone proteins, improved dopaminergic neuron integrity, and reduced α-synuclein pathology [175]. Abl-targeting TKIs have also been reported to modulate neuroinflammation and enhance amyloid and tau clearance in AD models [176,177]. However, translation to humans has been more challenging. Although early clinical studies suggested CSF/plasma biomarker changes consistent with altered proteostasis or protein clearance, later and more controlled studies have not demonstrated clear clinical efficacy, and dose-limiting toxicity, limited CNS exposure, and safety concerns remain major obstacles for chronic use in neurodegenerative populations [178,179]. Another TKI, masitinib, which targets KIT, Lyn, and Fyn kinases and was initially developed for mast-cell tumors and other malignancies, has progressed further clinically in AD. Phase II and III studies have reported slowed cognitive decline in subsets of patients with mild-to-moderate AD when used as add-on therapy to cholinesterase inhibitors [180]. However, these findings require cautious interpretation, as treatment effects may depend on patient selection, disease stage, dose, and tolerability. Masitinib’s proposed mechanism involves modulation of mast-cell and microglial activation, positioning it as an anti-neuroinflammatory strategy relevant to AD [180].

A distinct and increasingly influential repurposing strategy focuses on epigenetic regulators originally developed for cancer. Histone deacetylase inhibitors (HDACi), including vorinostat and panobinostat, reverse transcriptional repression through increased histone acetylation [181]. In AD and FTLD-spectrum tauopathies, chromatin dysregulation contributes to synaptic vulnerability and impaired neuronal resilience [137,182,183,184]. In preclinical models, HDAC inhibition restored synaptic/plasticity gene expression, improved synaptic function, and rescued memory deficits [137]. However, systemic toxicity and tolerability remain major barriers to clinical translation [185].

A major conceptual advance linking cancer biology to neurodegeneration is the recognition of cellular senescence as a shared but oppositely regulated mechanism. Senescence suppresses tumorigenesis but accumulates during aging and contributes to chronic inflammation through the SASP [186,187,188,189]. Senescent astrocytes, microglia, and oligodendrocyte progenitors have been identified in AD, PD, and FTLD-related models [111,113,190,191]. This has driven interest in senolytic therapies originally developed in oncology. The best-studied senolytic regimen combines dasatinib and quercetin [192,193]. Dasatinib targets pro-survival pathways upregulated in senescent cells, whereas quercetin inhibits complementary anti-apoptotic signaling networks [194,195,196]. Together, this combination reduces senescent-cell burden, suppresses SASP signaling, and improves tissue function in aging models. In AD models, senolytic clearance of senescent glial cells has been reported to reduce neuroinflammation and improve cognition [197]. However, evidence for cognitive benefit in humans remains limited, and translation to elderly neurodegenerative populations poses important challenges. These include the need for repeated or chronic administration, potential off-target effects, immunological consequences, drug–drug interactions, and tolerability concerns related to agents originally developed for oncology. Another potent senolytic is navitoclax (ABT-263), a BCL-2/BCL-XL inhibitor developed for small-cell lung cancer and hematologic malignancies. Navitoclax efficiently eliminates senescent cells and reduces neuroinflammatory markers in aging models. However, thrombocytopenia represents major obstacles for chronic use, particularly in elderly patients with neurodegenerative disorders [198]. Thus, senolytics remain promising but experimental therapeutic candidates rather than established disease-modifying treatments for AD or related dementias.

Finally, large-scale data-driven repurposing approaches integrating transcriptomics, network biology, and clinical datasets have identified additional oncology-derived therapeutic candidates [170]. One recent study identified irinotecan and letrozole as candidate AD therapeutics using disease-signature reversal analysis and electronic health-record data. This combination reduced amyloid and tau pathology and improved cognition in AD mouse models, while retrospective human analyses suggested reduced AD incidence among exposed patients [170]. These findings provide proof of principle for oncology-to-neurology drug-repurposing strategies.

## 2. Conclusions

In brief, this review examined in greater depth the relationship between specific types of malignant tumors and selected neurodegenerative disorders of the aging population, with the objective of identifying convergent and divergent biological pathways that may be relevant for clinical translation. This review highlights how targeting aging-related pathways including senescence and proteostasis may inform therapeutic development in both oncology and neurodegeneration. The inverse relationship between cancer and AD and related dementias represents one of the most intriguing observations in aging research. Population-based studies consistently report inverse associations between several malignancies and the risk of AD, LBD, and FTLD. At the molecular level, pathways hyperactivated in cancer, including PI3K/AKT/mTOR signaling, altered p53 function, enhanced DNA damage tolerance, and metabolic reprogramming, are often impaired in neurodegenerative disorders. Conversely, tumor-suppressive and stress response pathways that constrain proliferation may increase vulnerability of post-mitotic neurons to degeneration.

This review further emphasizes the paradoxical roles of aging-associated processes, particularly cellular senescence and loss of proteostasis, in driving divergent outcomes in oncogenesis versus neurodegeneration. Although studies adjusting for survival and competing-risk biases continue to support an inverse association, residual bias cannot be excluded [47]. Differences in medical surveillance and diagnostic ascertainment may also contribute to these findings. An unresolved question is whether specific cancer therapies influence AD risk or progression; while some observational studies suggest slower cognitive decline in subsets of cancer patients, treatment-related neurotoxicity (“chemo brain”) complicates interpretation. It remains unclear whether cancer reduces dementia risk, whether neurodegeneration constrains oncogenesis, or whether both reflect shared upstream aging mechanisms. Multiple molecular candidates, including PIN1, p53, IGF-1 signaling, telomerase, and DNA repair pathways, have been implicated; however, the causal mechanisms linking cancer and neurodegeneration remain incompletely understood. Caution is warranted because enhancing neuronal survival pathways may increase cancer risk, whereas inhibiting proliferative pathways could exacerbate neurodegeneration.

Future studies will likely need to address the need for longitudinal epidemiological analyses with rigorous adjustment for survival, detection, and competing-risk biases. Also, there is a need for more mechanistic studies in cellular and animal models to establish causality; integrated genetic, epigenomic, and transcriptomic analyses to identify common regulatory nodes; clinical trials targeting fundamental aging mechanisms such as senescence, DNA repair, and autophagy; and integration of these approaches within the geroscience framework.

As global populations continue to age, the burden of both cancer and neurodegenerative disorders will increase. Understanding how these disorders intersect may ultimately support integrated strategies for biomarkers, prevention, and disease-modifying therapeutics targeting fundamental aging mechanisms.

## Data Availability

No new data were created or analyzed in this study.
